# Development of an Aged Full-Thickness Skin Model Using Flexible Skin-on-a-Chip Subjected to Mechanical Stimulus Reflecting the Circadian Rhythm

**DOI:** 10.3390/ijms222312788

**Published:** 2021-11-26

**Authors:** Subin Jeong, Jisue Kim, Hye Mi Jeon, Kyunghee Kim, Gun Yong Sung

**Affiliations:** 1 Interdisciplinary Program of Nano-Medical Device Engineering, Graduate School, Hallym University, Chuncheon 24252, Korea; 21subin21@naver.com (S.J.); prtty_u5588@naver.com (J.K.); gpal1254@naver.com (H.M.J.); seoulhee92@naver.com (K.K.); 2 Integrative Materials Research Institute, Hallym University, Chuncheon 24252, Korea; 3 Major in Materials Science and Engineering, School of Future Convergence, Hallym University, Chuncheon 24252, Korea

**Keywords:** flexible skin-on-a-chip, circadian rhythm, periodic compressive stress

## Abstract

The skin is subject to both intrinsic aging caused by metabolic processes in the body and extrinsic aging caused by exposure to environmental factors. Intrinsic aging is an important obstacle to in vitro experimentation as its long-term progression is difficult to replicate. Here, we accelerated aging of a full-thickness skin equivalent by applying periodic mechanical stimulation, replicating the circadian rhythm for 28 days. This aging skin model was developed by culturing a full-thickness, three-dimensional skin equivalent with human fibroblasts and keratinocytes to produce flexible skin-on-a-chip. Accelerated aging associated with periodic compressive stress was evidenced by reductions in the epidermal layer thickness, contraction rate, and secretion of Myb. Increases in β-galactosidase gene expression and secretion of reactive oxygen species and transforming growth factor-β1 were also observed. This in vitro aging skin model is expected to greatly accelerate drug development for skin diseases and cosmetics that cannot be tested on animals.

## 1. Introduction

The skin, which protects the body from the external environment, is subject to both intrinsic and extrinsic aging processes. Intrinsic aging is caused by metabolic processes that occur in the human body, and extrinsic aging encompasses changes associated with long-term exposure to environmental factors such as ultraviolet (UV) rays [1,2]. Intrinsic aging may result from exposure to reactive oxygen species (ROS), which form as part of metabolic processes in the body, and which may become damaging when accumulated. Extrinsic aging occurs in response to harmful ROS formed by exposure to UV rays [3,4,5]. Under general conditions, the activity of receptor tyrosine kinase (RTK) on the surface of skin cells is suppressed by receptor protein tyrosine kinase (RPTP). However, when exposed to UV rays, ROS are produced by the skin and bind to RPTP. RTK is then phosphorylated to induce secretion of nuclear factor kappa-light-chain-enhancer of activated B cells (NF-κB) and activator protein-1 (AP-1) transcription factor. This transcription factor reduces the production of transforming growth factor (TGF)-β receptor and collagen, increases matrix metalloproteinase (MMP) gene transcription, and reduces collagen content [3,6,7]. 

The process of skin aging decreases epidermal thickness, barrier functionality, and replication ability [8]. In addition, the rate of keratinization of keratinocytes is reduced, thus delaying wound healing. In the dermis, the number of fibroblasts is decreased, collagen and elastic fibers are lost, and elasticity and dermal thickness are reduced. At the dermoepidermal junction, the boundary is flattened and the bond between the epidermis and dermis is weakened, so that even small stimuli may separate the epidermis from the dermis and induce damage [9]. To maintain the appearance of young and healthy skin, people use cosmetics and medicines to prevent or reverse skin aging. Many studies into skin aging and related treatment are thus ongoing [8,9,10].

β-galactosidase is a biomarker typically used to monitor aging. This enzyme hydrolyzes the β-glycoside bond between galactose and its organic moiety and can be detected at pH 6.0 in cultured cells when there are no proliferating cells present. The number and size of lysosomes increase in cultured cells with the number of passages, and the level of lysosomal-β-galactosidase also increases, as it is not expressed in active or dead cells [11,12]. β-galactosidase has been detected in elderly individuals and animals, and the expression of β-galactosidase displayed by senescent cells is a characteristic of biological aging [13,14]. Furthermore, when macrophages containing aged cells were transplanted into mice, the expression of β-galactosidase increased with time [15]. Therefore, β-galactosidase is the aging biomarker most commonly used because of its apparent specificity for aged cells: the more aged the cells are, the higher the expression of β-galactosidase [16]. 

Another aging biomarker used is proteasome 26S subunit, non-ATPase8 (PSMD8). As skin ages, the expression of the proteasome that removes damaged cells or misfolded proteins decreases. When proteasome activity decreases with age, abnormal proteins that interfere with the function of cells and tissues may accumulate. The proteasome has many subunits, and partial loss of PSMD8 from the epidermis interferes with proteasome function and reduces epidermal tissue thickness [17]. A previous study showed that the expression of PSMD8 from aged keratinocytes was 30% less than that from the skin of newborns [18]. In addition, because the epidermal and dermal layers show age-related changes, epidermal thickness, and the expression of filaggrin, keratin 10, involucrin, and fibronectin, among others, may be used to monitor aging.

The periodic pattern of daytime and nighttime created by the earth’s 24-hour rotation around its axis affects the development and functions of organisms. The circadian rhythm system comprises both central and peripheral clocks. Biological rhythmic regulators, also called peripheral clocks, are found in various tissues and cells, and are maintained by an autoregulatory feedback loop that includes transcription of the master regulatory genes circadian locomotor output cycles kaput (CLOCK) and brain and muscle ARNT-like 1 (BMAL1). This autoregulated feedback loop is formed when period circadian regulator 1 (Per1), Per2, Per3, cryptochrome circadian regulator 1 (Cry1), and Cry2 proteins accumulate in the cytoplasm [19,20]. In one study, fibroblasts, keratinocytes, and melanocytes were cultured and CLOCK gene expression was assessed via quantitative reverse transcription–polymerase chain reaction (qRT-PCR) [21]. The expression rate of the clock gene increased/decreased for 12 h, and the expression rate tended to decrease/increase during the next 12 h. The in vivo cycle of processes occurring in living organisms was shown to be similar to that occurring in cultured cells. The circadian rhythm is determined by environmental zeitgebers and internal clock rhythms. Zeitgebers include light, temperature, atmospheric conditions, and motion, among others [22]. Accordingly, a wrinkled skin model was previously developed by subjecting the tissue to a constant cycle of tensile stimulation for 12 h followed by 12 h without stimulation to create an environment similar to that in which chemical reactions take place in vivo [23]. 

Preclinical studies, which are essential for the development of new drugs and cosmetics, involve animal experimentation. However, cosmetic toxicity testing on animals was banned by the European Commission in 2013 because of cost, time, and ethical considerations, and the number of countries outside the European Union in which this is banned is increasing. “Skin-on-a-chip” was thus developed to replace animal experiments and create a model using human skin equivalents in vitro in three-dimensional (3D) culture to mimic the human physiological environment [24,25,26]. Cells are supplied with culture medium via a microfluidic channel in a static 3D environment. However, static experimental conditions differ from those in which physiochemical reactions take place in vivo as humans receive multiple stimuli both externally and internally [27]. Thus, “Skin-on-a-chip” cultivated skin tissue within microfluidic systems are capable of controlling many physical and chemical mediating conditions such as medium flow, mechanical force, and the inclination of biochemicals by imitating the 3D microenvironment of human skin [28]. According to the paper, it was confirmed that the epidermal–dermal interface of HSE-on-a-chip was preserved up to 4 weeks. Moreover, the HSE-on-a-chip was treated with doxorubicin for use in drug toxicity studies. The results showed that doxorubicin treatment induced spatial exfoliation along the epidermal–dermal interface into the basal layer. Therefore, it was suggested that such a skin-on-a-chip could also be used for drug toxicity studies [29]. Our research confirmed that providing media to a pumpless skin-on-a-chip system with gravity flow using 3D skin equivalents cultured on transwell inserts and microfluidic chips provided sufficient nutrients for cells to multiply and differentiate [24]. Furthermore, human skin equivalent models that use pumpless skin-on-a-chip systems with gravity flow are used as part of drug screening platforms to evaluate drug efficacy [24,30,31,32,33,34]. A previous study also confirmed that the thickness of the epidermal layer was increased by mechanical stretching [35]. However, this did have a tensile effect on 3D culture model. Studies have described the direct application of tensile stimulation to cells on a 2D culture model using piezoelectrically or electromagnetically actuating stretching devices, but compressive stimulation has not been previously applied to 3D culture models [24,36,37,38,39]. 

In this study, we used a flexible skin-on-a-chip (FSOC) and mechanical stimulus actuating system (MSAS). Mechanical compression stimulation that reflects circadian rhythms was applied to a 3D skin equivalent to produce an aging skin model. Aging markers were examined in a 28-day comparative culture experiment to demonstrate progression of the aging process occurring in the dermis and epidermis. Although many studies have been conducted on skin aging associated with UV exposure, no skin aging studies have investigated the equilibrium of 3D full-thickness skin equivalents subjected to mechanical compression associated with circadian rhythms. The use of this novel skin-on-a-chip aging model is expected to uncover new aging mechanisms that could be used for the screening and efficacy testing of new anti-aging substances. This is of great importance for the development of new drugs for the treatment of skin diseases and anti-aging cosmetics.

## 2. Results and Discussion

### 2.1. Contraction and Changes in Epidermal Thickness

**Figure 1:**
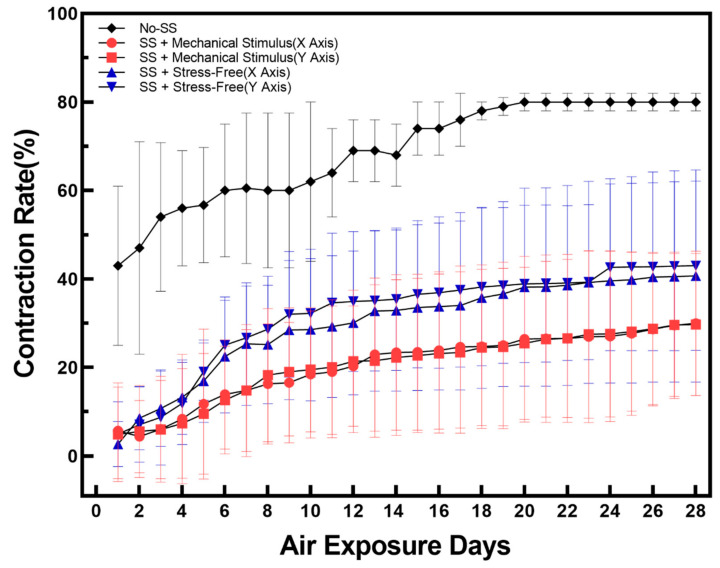
Variations of tissue contraction rate during 28 days of air exposure for three types of samples: No-SS means no sulfo-SANPAH treatment and no mechanical stimulus; SS + Mechanical Stimulus (*X* Axis) means sulfo-SANPAH treatment sample applied by along compressive stimuli direction; SS + Mechanical Stimulus (*Y* Axis) means sulfo-SANPAH treatment sample applied by along compressive stimuli direction; SS + Stress-free (*X*, *Y* Axis) means sulfo-SANPAH treatment sample without compressive stimuli. For each condition, the tissue contraction was calculated from eight chips and the average value was displayed. Error bars indicate standard error of the mean.

To minimize contraction, which is one of the biggest obstacles in covalently cultured full-thickness skin equivalents, the epidermis and dermis were treated with sulfo-sulfosuccinimidyl 6-(4′-azido-2′-nitrophenylamino)hexanoate (SANPAH), a crosslinker that firmly attaches collagen extracellular matrix (ECM) to the walls of the culture chamber. This decreased the contraction rate (Figure S1). As seen in Figure 1, sulfo-SANPAH treatment resulted in a 40% reduction in contraction over 28 days during the air-exposure incubation period, and the contraction rate was reduced by a further 10% when mechanical compressive stimulation was applied to sulfo-SANPAH treated FSOC (Figure S2). This decrease in contraction rate associated with mechanical compression stimulation is thought to be caused by a decrease in paracrine signaling between fibroblasts and keratinocytes [40,41], which can be considered to reflect accelerated cell aging. The experimental results of the ‘Secretion of Cytokines’ part proved that the contraction rate decreased due to the influence of paracrine signaling. 

**Figure 2:**
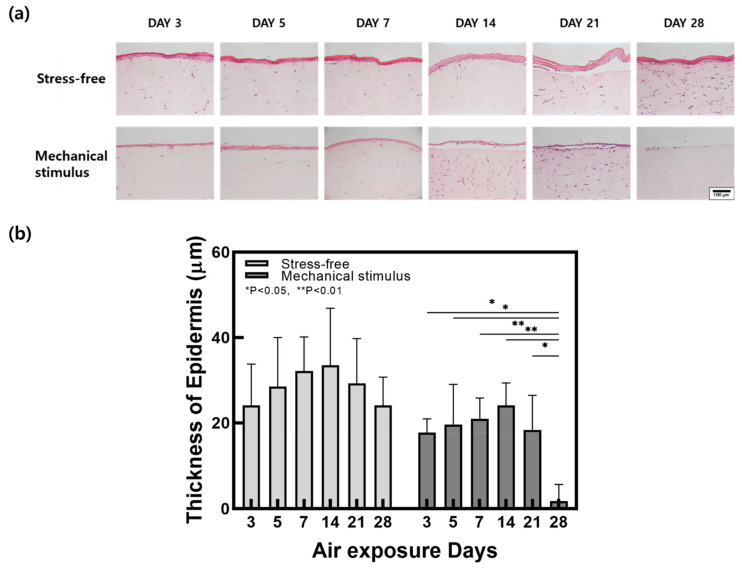
(**a**) H&E stained images and (**b**) epidermal thickness change in samples with and without compressive stimulation as a function of the air exposure days (Scale bar = 100 µm, *n* = 5, error bars = SEM, *, *p* < 0.05; **, *p* < 0.01).

Hematoxylin and eosin (H&E) was used to stain histological samples from the control group and the mechanical compression stimulation group. Changes in epidermal thickness are shown in Figure 2. Epidermal thickness increased in both groups until day 14 and showed a similar decrease thereafter. However, epidermal thickness was significantly decreased by compression stimulation. As skin thickness is reported to decrease with age, it is believed that compression stimulation caused accelerated aging in FSOCs [42]. 

### 2.2. Gene Expression

**Figure 3:**
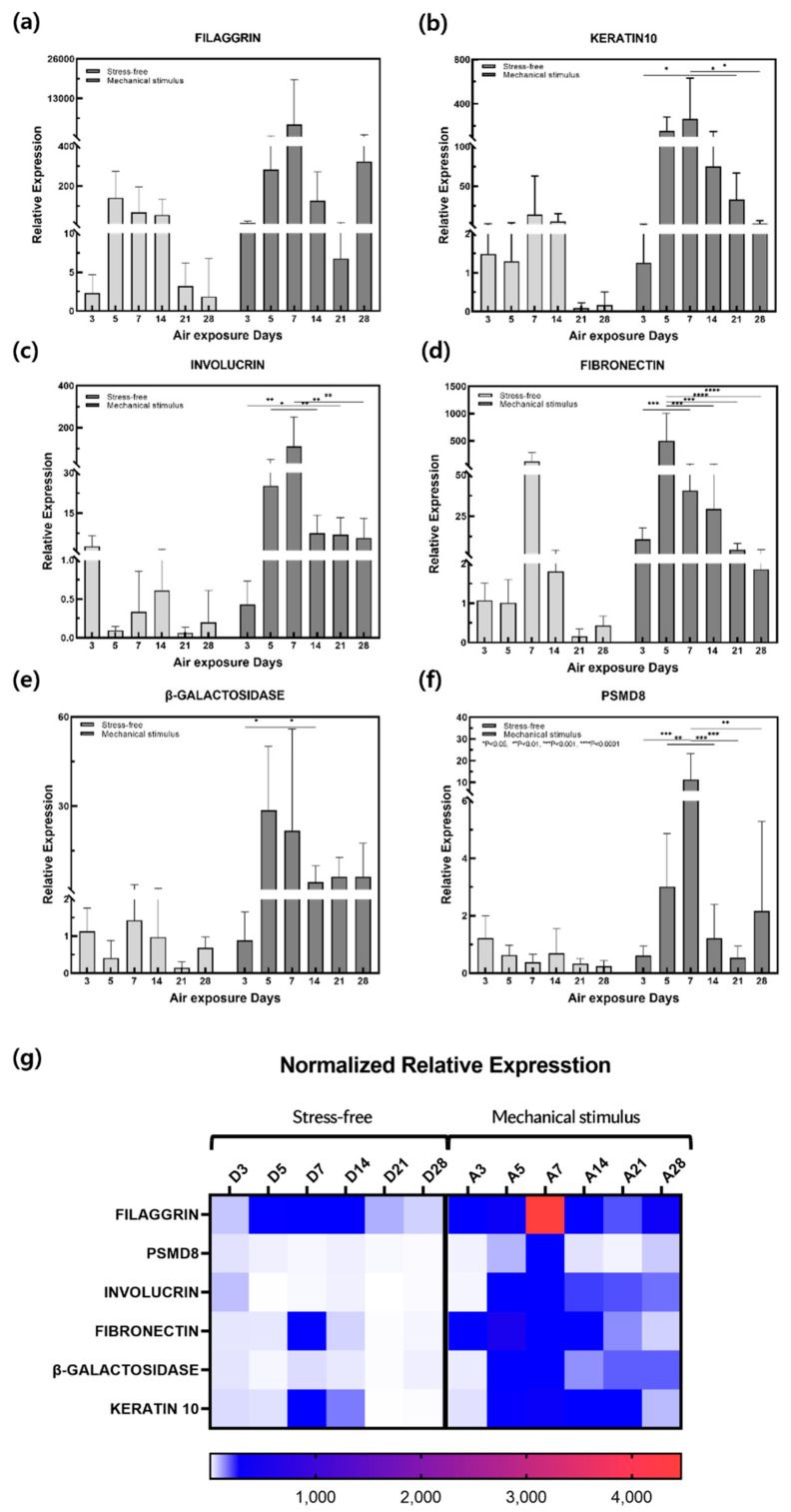
Relative gene expression of (**a**) filaggrin, (**b**) fibronectin, (**c**) involucrin, (**d**) keratin 10, (**e**) β-galactosidase, and (**f**) PSMD8 in samples with and without compressive stimulation as a function of the air exposure days. (**g**) Normalized relative expression for (**a**–**f**). (*n* = 5; *, *p* < 0.05; **, *p* < 0.01; ***, *p* < 0.001; ****, *p* < 0.0001).

The skin not only controls body temperature and immune function but also prevents the loss of water, electrolytes, and proteins from the body. Its structure consists of epidermal, dermal, and fat layers. The epidermis comprises the stratum corneum, stratum lucidum, stratum granulosum, stratum spinosum, and stratum basale. The dermis comprises the papillary and reticular layers [43]. Gene expression was measured via qPCR and the control and mechanical stimulation groups were compared (Figure 3). Keratin and filaggrin make up most of the proteins in the epidermis, which is the outermost layer of skin. In the stratum corneum, the outermost epidermal layer, filaggrin binds fibrous keratin tissues and strengthens the skin’s barrier function [44]. The stratum lucidum comprises two to three layers of epithelial cells. External application of keratin 10, one of the cytoplasmic components of these epithelial cells, maintains cell stability [45]. Filaggrin gene expression decreased in the control group over the culture period. In the mechanical stimulation group, expression increased on day 7 and again on day 28 (Figure 3a). Keratin 10 gene expression decreased slightly on day 5 in the control group but increased on days 7 and 14 before decreasing significantly. Keratin 10 gene expression was increased by mechanical stimulation until day 7 before decreasing on day 14. Overall, expression was significantly higher than that in the control group (Figure 3b). Involucrin is one of the components that make up the keratin cell membrane. It is synthesized faster than other structural proteins and also appears in the stratum granulosum and stratum spinosum. The stratum granulosum is the layer in which keratinocyte differentiation begins, and this thin layer of cells plays the most important role in the skin’s barrier function [46]. Involucrin gene expression decreased in the control group on day 5, increased on day 14, and then tended to decrease. Overall, expression was lower than that in the mechanically stimulated group. In this group, the expression increased sharply on day 7 in response to mechanical stimulation before decreasing steadily from day 14 (Figure 3c). The reticular layer occupies most of the dermis and is mainly composed of collagen, elastin, and glycoproteins. Collagen plays a significant role in supporting the skin, and elastin is important for skin elasticity. Glycoproteins affect cell–cell and fiber–fiber adhesions, and fibronectin is the most important adhesion protein [47]. Fibronectin gene expression increased significantly in the control group on day 7, although expression remained low overall. Expression also increased in the mechanical stimulation group from day 5 and decreased gradually over the rest of the culture period (Figure 3d). The highest expression level recorded was in the mechanical stimulation group, and the subsequent decrease observed was similar to that in the control group. 

β-galactosidase gene expression was significantly increased by mechanical stimulation from day 5 to 28 (Figure 3e). β-galactosidase is commonly used as a biomarker for aging as the number and size of lysosomes in cells gradually increase with age, resulting in increased lysosomal-β-galactosidase protein expression [8,9,48]. Therefore, overexpression of the β-galactosidase gene confirmed that aging was accelerated in response to mechanical stimulation. Expression of the proteasome, which removes damaged or misfolded proteins from cells, decreases with age and is induced by decreased expression of PSMD8 in certain proteasomal subunits [17]. Aging is associated with decreased PSMD8 expression in the epidermis and aging keratinocyte precursors also replicate at a lower rate [18]. PSMD8 gene expression increased sharply from day 7 in response to compression stimulation and then decreased significantly. This trend differed from the stable decrease observed in the control group (Figure 3f).

### 2.3. Protein Expression

**Figure 4:**
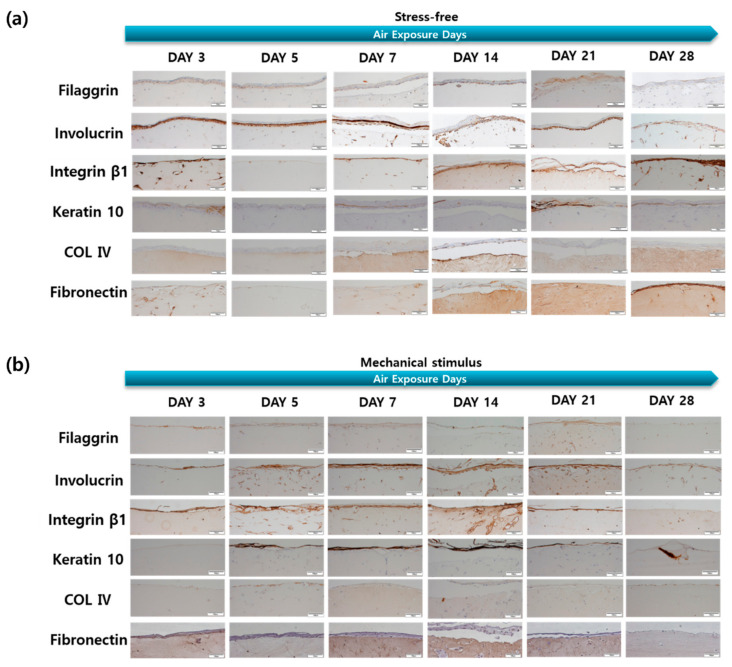
IHC stained images of the filaggrin, involucrin, integrin β-1, keratin 10, collagen IV, fibronectin for the samples (**a**) without and (**b**) with compressive stimulation for various air exposure days (Scale bar = 100 µm).

**Figure 5:**
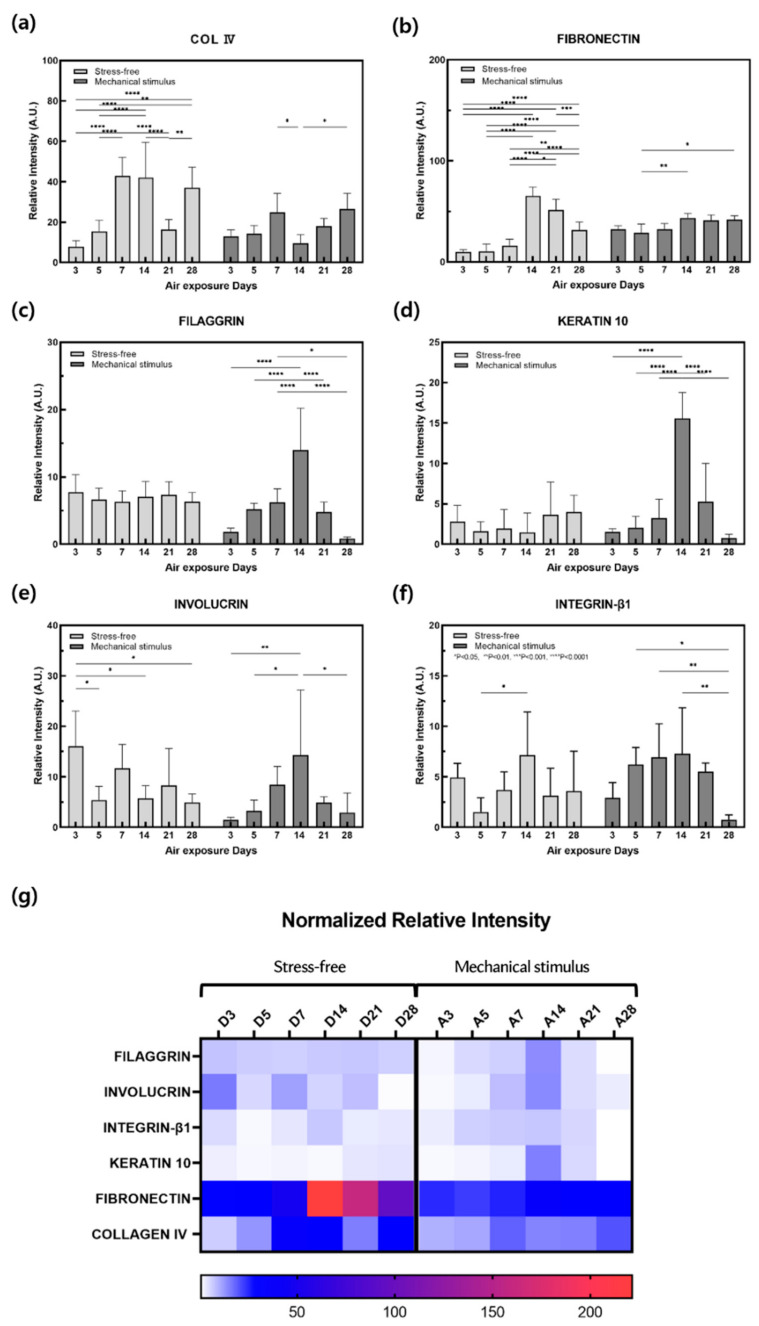
Quantitative analysis of IHC stained images of the (**a**) collagen IV, (**b**) fibronectin, (**c**) filaggrin, (**d**) keratin10, (**e**) involucrin, (**f**) integrin-β for the samples without and with compressive stimulation for various air exposure days. (**g**) Normalized relative intensity of (**a**–**f**). (*n* = 5; *, *p* < 0.05; **, *p* < 0.01; ***, *p* < 0.001; ****, *p* < 0.0001).

The effect of mechanical stimulation on protein expression levels in the dermal and epidermal layers was assessed via immunohistochemistry (IHC; Figure 4). Collagen IV (COL IV) is both the most important structural collagen of the basement membrane and entails key signaling potential, which is important for various physiological and pathological functions [49]. Collagen IV expression in the control group was high on days 7 and 14, decreased gradually, and then increased again on day 28 (Figure 5). In the control group, fibronectin expression increased significantly on day 14, but showed a gradual overall decrease. This may be associated with aging and may result from the reduced expression of two dermal proteins in response to mechanical stimulation during the culture period.

There were significant changes in the expression of filaggrin, keratin 10, involucrin, and integrin β-1 in the epidermal layer in the control group. Involucrin showed an overall tendency to decrease, although this was significant on days 5 and 14. The amount of integrin β-1 in keratinocytes decreases with age and in the elderly, as keratinocytes have a low integrin β-1 content, low self-renewal capacities, and altered adhesion properties [50]. Integrin β-1 decreased from day 5, increased again, and finally decreased on day 21. The expression of these four epidermal proteins tended to decrease on day 21 and then decreased significantly on day 28, while increasing up to day 14 in response to mechanical stimulation. This may be due to an increase in keratin 10 and involucrin gene expression and a decrease in protein expression after day 7. Furthermore, these results are thought to indicate that mechanical stimulation accelerated aging more rapidly after day 14, as epidermal thickness and the expression of filaggrin, keratin 10, involucrin, and integrin β-1 remained the same after this day. Therefore, expression behaviors such as filaggrin, keratin 10, involucrin, and fibronectin can also be considered as evidence of aging.

### 2.4. ROS Production and Cytokine Secretion 

**Figure 6:**
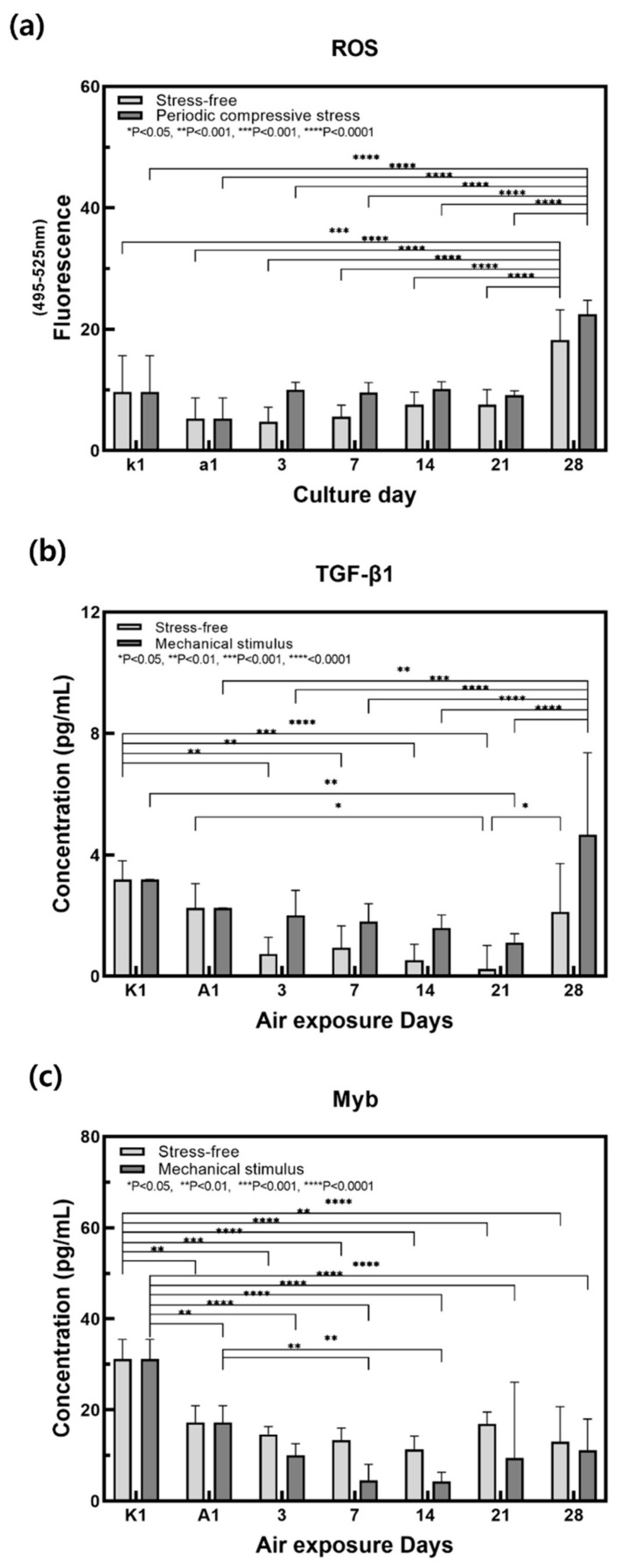
Variation of (**a**) ROS, (**b**) TGF-β1, (**c**) Myb secretion for the samples without and with compressive stimulation for various air exposure days. (*n* = 3; *, *p* < 0.05; **, *p* < 0.01; ***, *p* < 0.001; ****, *p* < 0.0001).

The ROS production was measured in the culture medium according to the control group and the mechanically stimulated group (Figure 6a). ROS production was increased by mechanical stimulation. ROS are produced through normal intracellular activation but may accumulate if there is an imbalance in the antioxidant reaction that removes it. ROS can then have damaging effects on DNA, proteins, and lipids, and is known to induce aging [51,52]. The production of ROS was particularly high on day 28. It is suggested that the antioxidant defense function that removes active oxygen decreased and thus ROS production increased sharply when subjected to periodic mechanical stimulation over a long period of time. Therefore, mechanical stimulation is thought to accelerate the process of aging.

When ROS are generated intracellularly, it binds to RPTP, which suppresses the activation of RTK on the cell surface and induces NF-κB and AP-1 activity. NF-κB increases MMP gene transcription and suppresses collagen production, and AP-1 reduces TGF-β receptor expression and suppresses subsequent collagen production [3,6]. As shown in Figure 6b, the concentration of TGF-β1 increased in response to mechanical stimulation, and this increase was noticeable on day 28. TGF-β receptor expression was reduced in response to an increase in ROS, and high concentrations of unbound TGF-β1 were therefore detected in the medium. Collagen production was consequently suppressed and the process of aging was accelerated.

Myb concentration was measured in the culture medium in the control group and mechanical stimulation group (Figure 6c). Myb secretion decreased in response to mechanical stimulation. Myb is secreted by keratinocytes, which communicate with each other via cell–cell interactions and the release of paracrine factors such as TGF-β1 from fibroblasts [41]. Studies have shown that decreased TGF-β1 secretion induced Myb deficiency in keratinocytes, which reduced Myb-induced collagen I deficiency [53]. Therefore, it is thought that mechanical stimulation reduced the secretion of Myb from keratinocytes and collagen I production from fibroblasts, thus reducing contraction.

**Figure 7:**
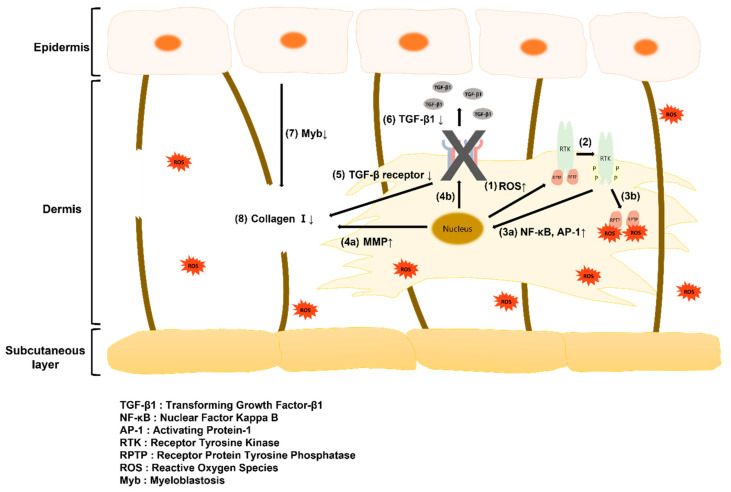
Schematic illustration of the paracrine signaling between fibroblasts and keratinocytes in the skin tissue: (1) Increase in ROS secretion from the nucleus by compressive stimulation; (2) RTK phosphorylation; (3a) increase in secretion of NF-κB and AP-1 delivered to the nucleus by phosphorylated RTK; (3b) RTK phosphorylation by binding of RPTP and ROS; (4a) Increase in MMP secretion from the nucleus; (4b) Signaling to decrease TGF-β receptor in the nucleus; (5) Decrease in TGF-β receptor; (6) Decrease in TGF-β1 binding to TGF-β receptor; (7) Decrease in Myb secretion; (8) Decrease in collagen I in the dermis layer. Three possible pathways for skin aging: (1) ➔ (2) ➔ (3a) ➔ (4a) ➔ (8), (1) ➔ (2) ➔ (3a) ➔ (4b) ➔ (5) ➔ (8), (1) ➔ (2) ➔ (3a) ➔ (4b) ➔ (5) ➔ (6) ➔ (7) ➔ (8).

To investigate the mechanisms underlying mechanical stimulus-associated aging, a cytokine assay was performed. The results of this study may be explained by the aging mechanism depicted in Figure 7, as ROS and TGF-β1 levels increased and Myb level decreased in response to mechanical stimulation. Mechanical stimulation promotes intracellular activity and increases the production of ROS, which bind to RPTP on the cell surface to cause RTK phosphorylation and induce NF-κB and AP-1 expression. This reduces TGF-β receptor expression, suppresses collagen production, increases MMP gene transcription, and reduces collagen content. Dermal collagen I deficiency reduces Myb secretion from the stratum corneum. Therefore, periodic mechanical stimulation increased ROS secretion and decreased TGF-β1 receptor production, resulting in dermal collagen I deficiency and a subsequent decrease in Myb secretion. It is thought that aging progressed through this mechanism leads to aging-related observations such as a reduction in the contraction of the full-thickness skin equivalent and epidermal thickness and an increase in β-galactosidase gene expression, a marker of aging.

## 3. Materials and Methods

### 3.1. Flexible Skin-on-a-Chip (FSOC)

**Figure 8:**
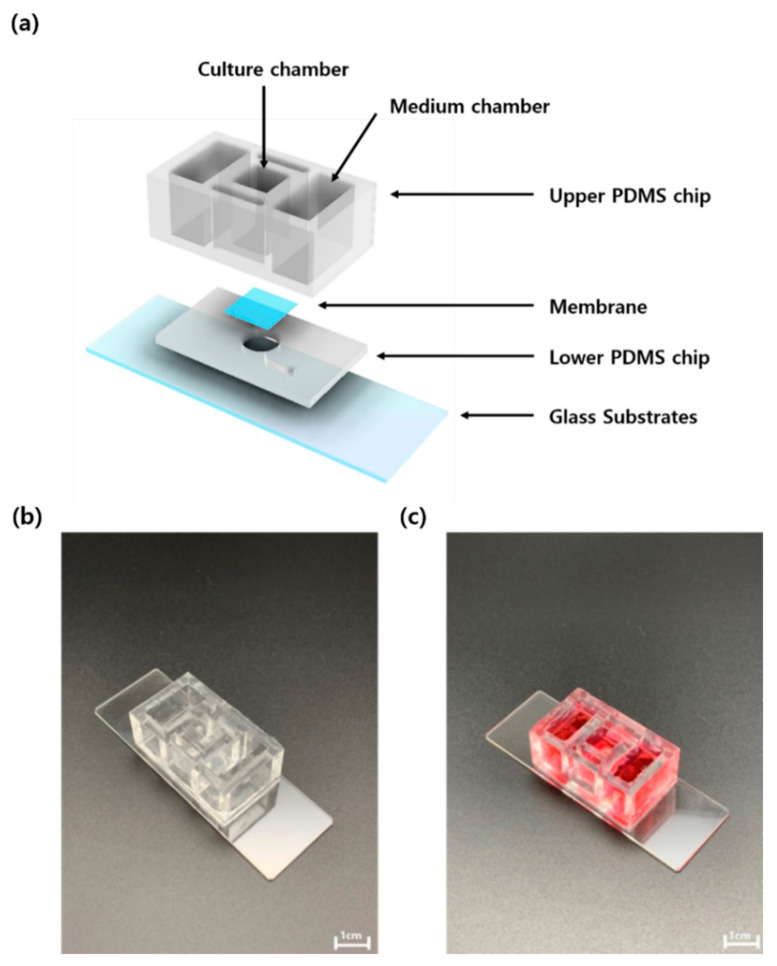
Schematic diagram of (**a**) the structural description of flexible skin-on-a-chip (FSOC), (**b**) actual photograph of FSOC and (**c**) actual photograph when medium is put in FSOC.

An upper polydimethylsiloxane (PDMS) (K-1 solution, Gwangmyeong, Korea) chip was manufactured by preparing a solution of PDMS using a 30:1 ratio of silicone elastomer base:silicone elastomer curing agent to confer flexibility. This was cured in an oven at 80 °C for 2 h. The lower PDMS chip was produced using a patterned master mold (width: 200 µm, height: 150 µm high) using a 10:1 ratio of silicone elastomer base:silicone elastomer curing agent. This was hardened in an oven at 80 °C for 1 h. The FSOC comprises an upper PDMS chip and a lower PDMS chip, between which a porous member (pore size: 0.4 µm; Corning Inc., New York, NY, USA) is placed (Figure 8). There is an 8-mm^2^ culture chamber in the center of the FSOC, which is used to cultivate cells in medium supplied through two medium chambers in the bottom membrane. Both medium chambers are filled with medium circulating through the lower channel [23,30,31,32,33,34].

### 3.2. Crosslinker Processing

**Figure 9:**
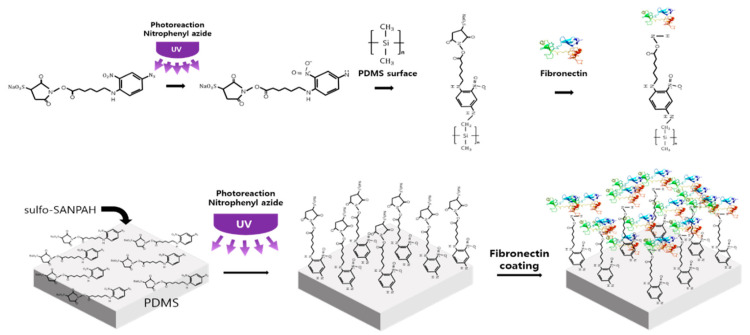
Schematic diagrams for a binding process of sulfo-SANPAH, Fibronectin and PDMS.

A photo-crosslinker was used to minimize sample contraction [54,55]. According to various papers using Sulfo-SANPAH, it has been proven that there is no effect on cells in the case of long-term experiments [54,55,56]. A high-power UV curing device (JHCI-051BS-V2; Jueun UV Teq Anyang, Korea) was used after filling the FSOC cell chamber with 10 mM sulfo-SANPAH (Proteo Chem, Hurricane, UT, USA; Figure 9). The device is used to expose for 45 min under a high-power UV (wavelength 365 nm) of 400 W at a distance of about 15 cm from the PDMS surface. During this process, a nitrophenyl azide reaction occurs and the sulfo-SANPAH molecules become perpendicular and bind to the PDMS surface. Next, the cell chamber was then cleaned with PBS, filled with a fibronectin solution (0.66 mg/mL Corning Inc., New York, NY, USA), reacted to the cell chamber for 3 h at 4 °C, then cultured in the cell. During this process, fibronectin binds to the amine group at the end of the sulfo-SANPAH attached to the PDMS surface. The bound fibronectin then binds strongly to the 3D culture support, collagen, and prevents contraction during culture.

### 3.3. Cell Culture

Primary human fibroblasts (FBs, Bio Solution Co Ltd. Seoul, Korea) were cultured in Dulbecco’s Modified Eagle’s Medium (DMEM) (gibco, New York, NY, USA) supplemented with 10% fetal bovine serum and 1% penicillin–streptomycin. Primary human keratinocytes (KCs, Bio Solution Co Ltd., Seoul, Korea) were cultured in keratinocyte growth medium (KGMTM, Lonza, Basel, Switzerland). All cultures were incubated at 37 °C in an incubator under 5% CO_2_, and all cells were used between passage numbers 4–7.

**Figure 10:**
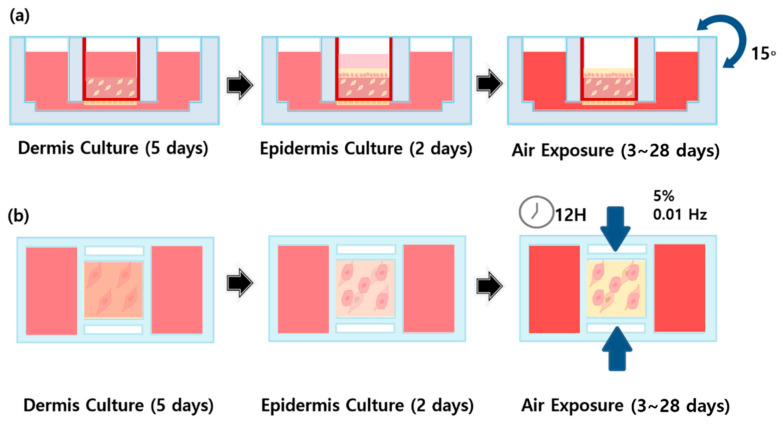
Schematic diagram of (**a**) FSOC 3D culture process using gravity flow system, (**b**) the 3D culture process in which periodic compressive stimulation is applied for 12 h using a mechanical stimulus actuating system (MSAS) and maintained in a non-stimulation state for 12 h.

The dermal layer was mixed with a type I collagen solution (Corning Inc., New York, NY, USA) and 5.0 × 10^5^ cell/mL fibroblasts and cultured in Dulbecco’s modified Eagle medium (DMEM; Gibco, New York, NY, USA) for 5 days (Figure 10). Next, 1.0 × 10^6^ cell/keratinocytes were attached to the dermal layer, and the epidermal layer was formed using KGMTM and DMEM over 3 days. During the air exposure phase (air liquid interface exposure), in which cells are directly exposed to the air to induce differentiation [57,58,59], samples in the mechanical stimulus group were subjected to alternating periods of compression (12 h, 5% compressive strain at 0.01 Hz) and no compression (12 h) for 28 days. As the initial conditions of the mechanical stimulus, 3%, 5%, 7%, 10% compressive strain and 0.01 Hz, 0.005 Hz were set for the experiment. As a result of the experiment, under the conditions of 7% and 10% compressive strain, heat occurred in the motor of the machine during the experiment and collagen was melted by heat conduction. It was also confirmed that 0.01 Hz controlled more tissue contraction than 0.005 Hz. Therefore, we adopted 5% compressive strain, 0.01 Hz as the final condition of the mechanical stimulus. Control samples were turned every 30 s at an angle of 15° for 28 days. During this period, samples were cultured in E-media comprising DMEM/Ham’s F12 (Gibco), 10 ng/mL epidermal growth factor (EGF)-1, 0.4 µg/mL hydrocortisone, 5 µg/mL insulin, 5 µg/mL transferrin, 2 × 10^−11^ M 3,3,5-triiodo-L-thyonine sodium salt, 10^−10^ M cholera toxin, 10% (*v*/*v*) fetal bovine serum (FBS), and 1% penicillin/streptomycin.

### 3.4. Gravity-Flow System

In 3D cell culture, a gravity-flow system is used to mimic the in vivo environment [24,60]. Flow is generated in the direction in which gravity acts on the culture solution, and the culture medium can thus be efficiently supplied. The gravity-flow system comprises a computer, a motor, and a stage, and can be used to control the time and angle. In this study, it was operated at 15° for 30 s and was continuously operated during the air exposure period. Each air exposure period was 3 days, 5 days, 7 days, 14 days, 21 days, and 28 days, and 8 chips were used per condition.

### 3.5. Mechanical Stimulus Actuating System (MSAS)

**Figure 11:**
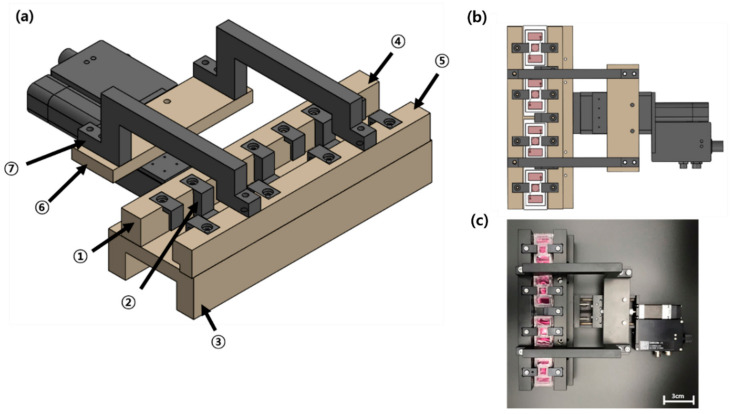
Schematic diagrams (**a**,**b**) and actual photograph (**c**) of the mechanical stimulus actuating system: ① Rack, ② Suspension, ③ Bottom plate, ④ Fixed top plate, ⑤ Moving top plate, ⑥ Bridge plate, ⑦ Bridge.

The driving system used to apply compression to the FSOC comprised seven components (Figure 11): a rack on which four chips were hanged, a suspension to prevent chips from floating or being dragged, a bottom plate on which to place chips, a fixed top plate for assembling the rack and suspension, a moving top plate that transmits the linear reciprocating motion of a linear motor, a bridge plate connecting the motor and the moving top plate, a bridge connecting the moving top plate part and the bridge plate part. Most of the components that were needed to block the thermal conduction of the linear motor were made of polyoxymethylene acetals with a very low thermal conductivity of 0.31 W/mK, while others were made of a lightweight alloy (Al6061;180 W/mK).

The drive motor uses a linear motor (Zaber, Vancouver, BC, Canada) and the Zaber Console Program provided by the manufacturer was used to apply the compression. The chips placed on the bottom plate are inserted into a fixed top plate and a moving top plate that connect each suspension to a rack. A moving top plate is connected to a bridge and a bridge plate. A linear motor operates by connecting a bridge and a bridge plate. Then, the motor moves stretched, and the force is transmitted to the bridge, bridge plate, and moving top plate fixed to the motor, and compression is applied to the chip. 

The circadian rhythm is determined by environmental factors such as light, temperature, and atmospheric conditions that accompany internal clock rhythms. Here, we considered kinetic factors. We therefore used eight chips undergoing 5% compressive strain at 0.01 Hz for 12 h and remaining inert for the next 12 h. This cycle was maintained for a defined period of air liquid interface exposure to mimic the circadian rhythm and related in vivo processes such as chemical reactions occurring in living organisms [24,60]. Each air exposure period was 3 days, 5 days, 7 days, 14 days, 21 days, and 28 days, and 8 chips were used per condition.

### 3.6. Hematoxylin and Eosin (H&E) Staining

The skin equivalent cultured on an FSOC was fixed using 4% formaldehyde and then paraffinized. After 12 h, an automatic tissue processor (Leica Biosystems, Wetzlar, Germany) was used to perform dehydration and clearing. This process allowed paraffin to penetrate the tissue and was maintained for 24 h. Samples infiltrated with paraffin were incubated in a refrigerator for 1 h, and then cut into 4–6-μm sections using a microtome. The tissue sections were brought into contact with heat to smooth out wrinkles, floated on water, and removed from the water using a slide to achieve complete adhesion. Slides were placed in an oven at 60 °C for 1 to 2 h to melt the paraffin, before deparaffinization was performed using xylene. Samples were stained with H&E and visualized using an optical microscope (Olympus IX73, Olympus, Tokyo, Japan).

### 3.7. Contraction Rate Analysis

The contraction rate is measured by taking a tissue picture every day and substituting it into the equation (Equation (1)). $L_{C}$ is the length of the chamber and $L_{T}$ is the length of the tissue.

(1) $$\frac{L_{C}\;-L_{T}\;}{L_{C}\;}\times 100\%$$

### 3.8. Immunohistochemistry (IHC)

After preparing slides using the method described for H&E staining, samples were incubated with the primary antibody and incubated the sections with horseradish peroxidase (HRP)-conjugated secondary antibody and visualized them using 3,3-diaminobenzidine (DAB) to detect the protein of interest [61]. One sample for each condition was divided into 5 fields under a microscope and expression intensities were analyzed using the open-source software ImageJ (FIJI). Each mean of the five values was compared relative. Primary antibodies used for IHC were Filaggrin (ab81468) (Abcam, Cambridge, UK), Fibronectin (ab32419) (Abcam), Involucrin (ab53112) (Abcam), Integrin β-1 (ab24693) (Abcam), Collagen IV (ab6586) (Abcam), Cytokeratin 10 (ab76318) (Abcam).

### 3.9. Quantitative Reverse Transcription–Polymerase Chain Reaction (qRT-PCR)

For qRT-PCR analysis, five full-thickness skin equivalent samples out of eight chips were used for each culture day. TRIzolTM (Thermo Fisher Scientific, Waltham, MA, USA) was used to extract mRNA from tissue samples. The extracted mRNA was quantified using a SpectraMax M2 Microplate Reader (Molecular Devices, San Jose, CA, USA), and was then treated with amfiRivert cDNA Synthesis Platinum Master Mix (GenDEPOT, Barker, TX, USA) to synthesize cDNA. qPCR was performed using a LightCycler^®^ 480 Instrument II (Roche, Basel, Switzerland) using LightCycler^®^ 480 SYBR Green I Master (Roche). Gene expression levels were analyzed by the ΔΔC_T method. GAPDH was used in the housekeeping gene used for qRT-PCR, and each primer pairs used for qRT-PCR are listed in Table 1.

### 3.10. Culture Medium Assay

Culture medium, which was replaced every 24 h, was collected from three chips in each of the control group and the mechanical stimulation group and stored at −20 °C. Enzyme-linked immunosorbent assay (ELISA) kits for TGF β-1 (BioVision, Milpitas, CA, USA), Myb (LSBio, Seattle, WA, USA), and intracellular ROS (fluorometric; Sigma–Aldrich, St Louis, MO, USA) were used according to the manufacturers’ instructions.

### 3.11. Statistical Analysis

Data are expressed as the mean ± standard deviation (SD). Statistical analysis was performed using Prism. Statistical significance was determined using a two-way ANOVA. A *p* value < 0.05 was considered statistically significant. Different significance levels (*p* values) are indicated by asterisks.

## 4. Conclusions

In this study, an in vitro full-thickness skin equivalent was used to develop an FSOC and MSAS cultured over 28 days to solve the problem of contraction, a major obstacle to in vitro skin models, using a photo-crosslinker and a mechanical stimulation environment reflective of circadian rhythms. The process of aging was evidenced by reduced contraction of the full-thickness skin equivalent subjected to mechanical stimulation, decreased epidermal layer thickness, and an increased β-galactosidase gene expression. Dermal expression of collagen IV and fibronectin decreased in aging samples in response to mechanical stimulation, and the expression of epidermal filaggrin, keratin 10, involucrin, and integrin β-1 genes increased approximately 7 days earlier, a trend also observed with regard to protein expression. 

Here, we created an aged full-thickness skin equivalent model that uses mechanical stimulus reflective of the circadian rhythm. This model will be useful for conducting in vitro drug efficacy assessments and investigating new cosmetics, as these cannot be tested on animals. This work paves the way for dramatically shortening the development period for novel therapeutic agents for skin diseases. It is ideal to implement an aging model with the same skin model as the human body by culturing all the cells that make up the skin of the human body at the same time. In this study, there is a limitation in that it is simple compared to the actual skin tissue as it is a skin equivalent fabricated under the conditions possible with the current co-culture technology. In the future, through many follow-up studies, it is considered necessary to produce an aging skin model based on a skin model in which skin tissue components such as immune cells, capillaries, peripheral nerve cells, hair follicles, and sweat glands are added.

## Figures and Tables

**Table 1:** List of primer sequences used for qPCR.

	Forward	Reverse
GAPDH	5′-CTCCTCTGACTTCAACAGCG-3′	5′-GCCAAATTCGTTGTCATACCAG-3′
Filaggrin	5′-GGAGTCACGTGGCAGTCCTCA-3′	5′-GGTGTCTAAACCCGGATTCAC-3′
PSMD8	5′-ATGTACGAGCAACTCAAGGG-3′	5′-CTGTGGTTGGCAAGAAGTTG-3′
Involucrin	5′-CCGCAAATGAAACAGCCAACTCC-3′	5′-GGATTCCTCATGCTGTTCCCAG-3′
Fibronectin	5′-CTGAGGGCAGAAGAGACAAC-3′	5′-TCATGCTGCTTATCCCACTG-3′
β-galactosidase	5′-AATCAAGACCGAAGCAGTGG-3′	5′-GGCATCATAGTCGTAGCTGG-3′
Keratin10	5′-CCGGAGATGGTGGCCTTCTCTCT-3′	5′-GGCCTGATGTGAGTTGCCATGCT-3′

## Supplementary Materials

The following are available online at https://www.mdpi.com/article/10.3390/ijms222312788/s1.
