# Supplementary material for: Development of an Aged Full-Thickness Skin Model Using Flexible Skin-on-a-Chip Subjected to Mechanical Stimulus Reflecting the Circadian Rhythm
Source: Int J Mol Sci. 2021 Nov 26;22(23):12788. doi: 10.3390/ijms222312788 (PMC8657468; doi:10.3390/ijms222312788)
Supplement: Supplementary file 1 [file ijms-22-12788-s001.zip › ijms-1463942-supplementary/Aging Skin Model supplement - 211030.pdf]

Supplementary information

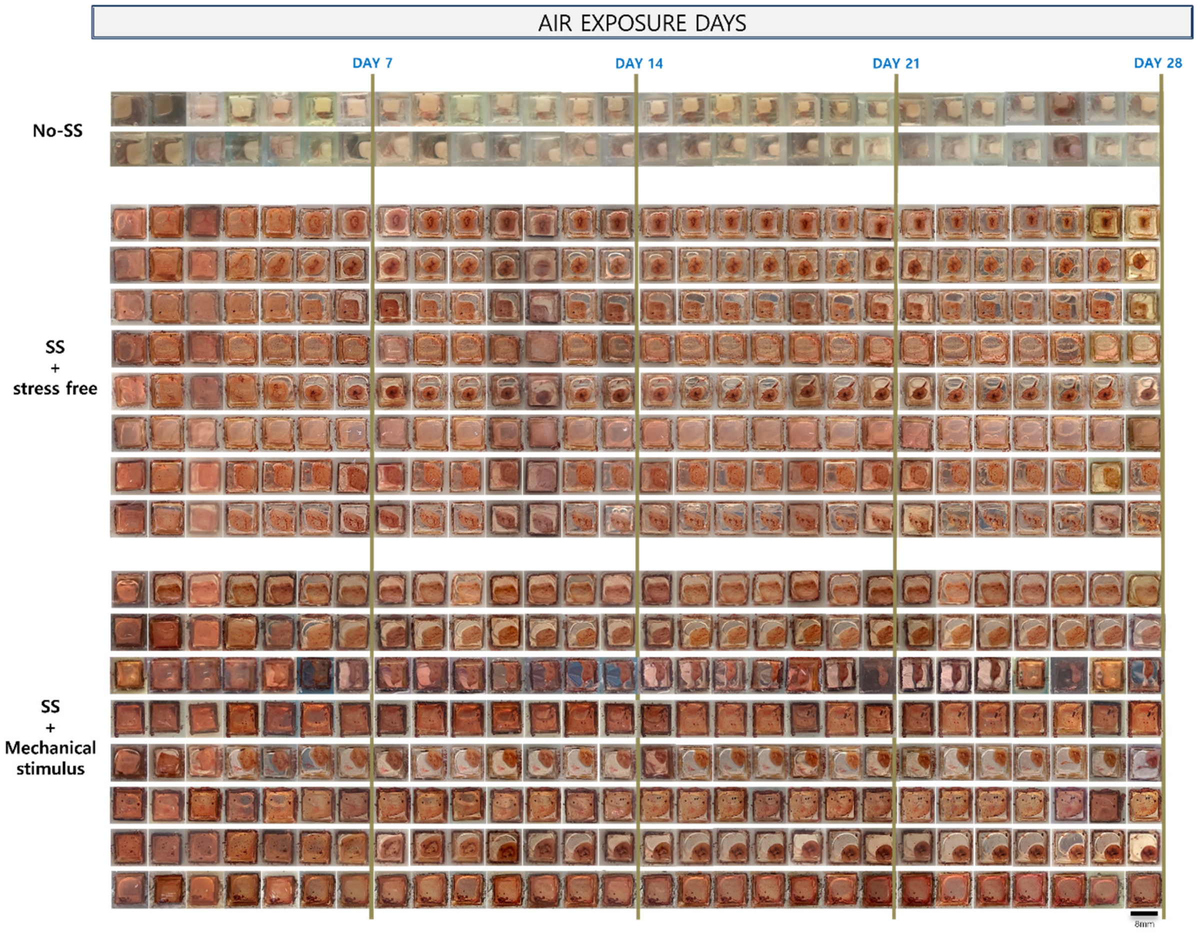

Figure S1. Photographs of changes in tissue contraction rate during 28 days of air exposure for three types of samples. (Scale bars = 8mm) No-SS means no sulfo-SANPAH treatment and no mechanical stimulus, SS+Stress-free means sulfo-SANPAH treatment sample without compressive stimuli, SS+Mechanical Stimulus means sulfo-SANPAH treatment sample with compressive stimuli.

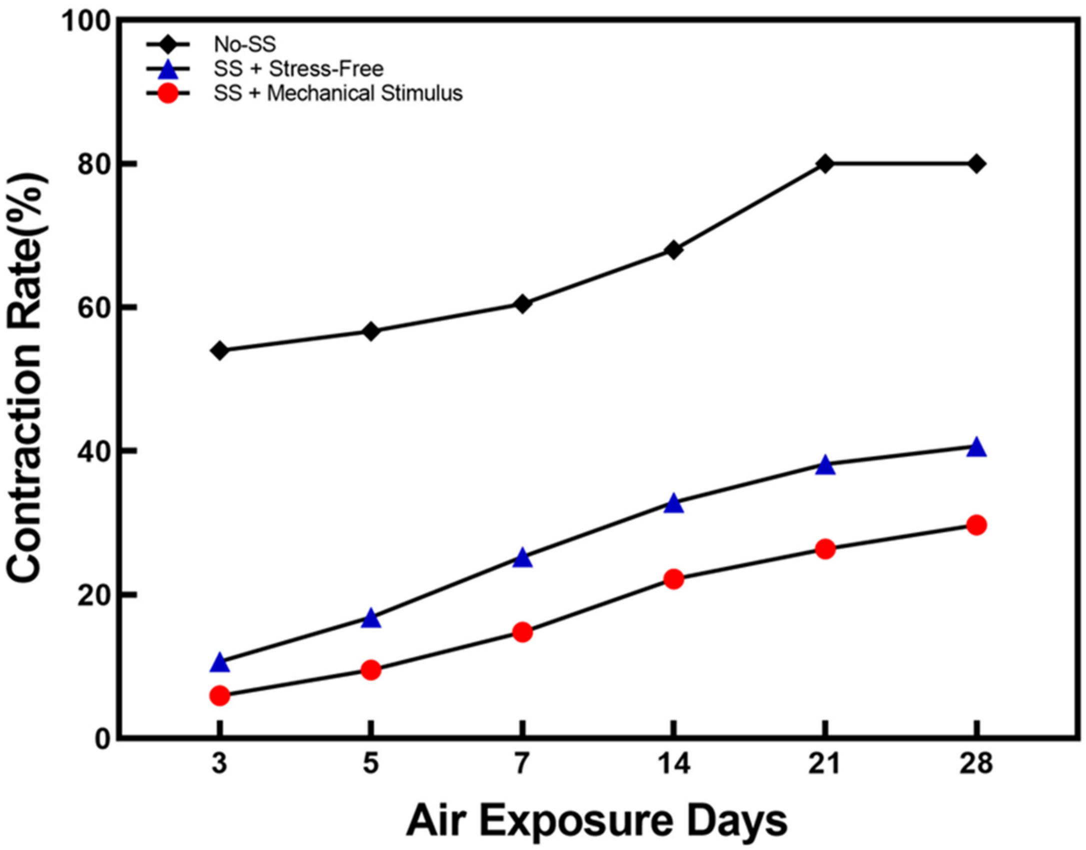

	(No-SS) – (SS+Stress-free) (%)	(SS+Stress-free) – (SSA+Mechanical stimulus) (%)
3 Days	43.28977	4.744321
5 Days	39.80606	7.357955
7 Days	35.20929	10.51381
14 Days	35.12879	10.66585
21 Days	41.82576	11.79822
28 Days	39.33674	10.94913

Figure S2. Difference in contraction rate by air exposure period according to sulfo-SANPAH and mechanical stimulus treatment conditions.
